# Comparison of skin-sparing mastectomy using LigaSure™ Small Jaw and electrocautery

**DOI:** 10.1186/s12957-017-1199-z

**Published:** 2017-07-14

**Authors:** Young Woo Chang, Hwan Soo Kim, Seung Pil Jung, Sang Uk Woo, Jae Bok Lee, Jeoung Won Bae, Gil Soo Son

**Affiliations:** 1 Department of Surgery, Korea University College of Medicine, Seoul, Republic of Korea; 2 Department of Breast Endocrine Surgery, Korea University Medical Center, 123, Jeokgeumro, Danwongu, Ansan, Gyeonggi 15355 Korea

**Keywords:** Skin-sparing mastectomy, Energy devices, Electrocautery, LigaSure™ Small Jaw

## Abstract

**Background:**

Skin-sparing mastectomy (SSM) is increasingly used in patients with breast cancer. We compared the differences between use of electrocautery and LigaSure™ Small Jaw in patients with breast cancer who underwent SSM.

**Methods:**

Between January 2012 and December 2015, 81 patients with breast cancer who underwent SSM were selected and were divided into the electrocautery group and the LigaSure™ Small Jaw group based on the devices that were used. Clinicopathological characteristics, body mass index, operative time, and weight of removed breast were obtained from medical records. Total amount and days of drain use, until removal, and postoperative skin necrosis, requiring debridement, were also analyzed.

**Results:**

The study population consisted of 50 patients in the electrocautery group and 31 in the LigaSure™ Small Jaw group. The latter group has significantly shorter operative time (117.5 ± 16.9 vs. 104.0 ± 23.6 min, *P* = 0.004). The mean total volume of drainage was less (805 ± 278 vs. 694 ± 131 mL, *P* = 0.017) and mean duration of drainage was also significantly shorter in the LigaSure™ Small Jaw group (11.3 ± 2.5 vs. 10.1 ± 2.0 days, *P* = 0.029).

**Conclusions:**

The use of LigaSure™ Small Jaw during skin-sparing mastectomy shortened the operative time and duration of drainage and reduced the total volume of drainage.

## Background

Skin-sparing mastectomy (SSM), including nipple-sparing mastectomy (NSM), has emerged as an alternative to standard mastectomy and is known to be oncologically safe, with improved cosmetic results and quality of life for patients with breast cancer [1–3]. Because of this, SSM has become increasingly more common in recent years and the inclusion criteria have also been expanded [4]. However, SSM is technically more difficult and time consuming, since it has to be performed through a smaller incision than that used in standard mastectomy.

Electrocautery is the most commonly used surgical device for dissection and hemostasis in standard mastectomy; however, its wide thermal spread may lead to flap necrosis, wound infection, and prolonged drainage [5]. Moreover, electrocautery in SSM may not control minor bleeding properly since the operative view is limited. Some studies showed that an electrothermal bipolar vessel-sealing system could shorten the operative time and decrease blood loss and drainage volume as compared to the results with electrocautery [6, 7].

**Fig. 1:**
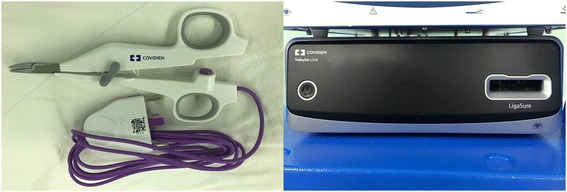
LigaSure™ Small Jaw and Valleylab™ LS10 Generator

LigaSure™ (Covidien, CO, USA) is an electrothermal bipolar vessel-sealing system that provides hemostasis by creating a seal using pressure and electrothermal energy to change the structure of the vessel walls and surrounding tissues. The LigaSure™ Small Jaw is a new instrument with a cutting blade and is often used for thyroid surgery (Fig. 1). This new energy instrument can be used for cutting as well as ligation of blood vessels up to 7 mm in diameter [8, 9].

To the best of our knowledge, few studies have compared the use of electrocautery and LigaSure™ Small Jaw in breast cancer patients undergoing SSM. Thus, this study aimed to compare intraoperative and postoperative outcomes using conventional electrocautery and LigaSure™ Small Jaw in patients undergoing SSM.

## Methods

**Fig. 2:**
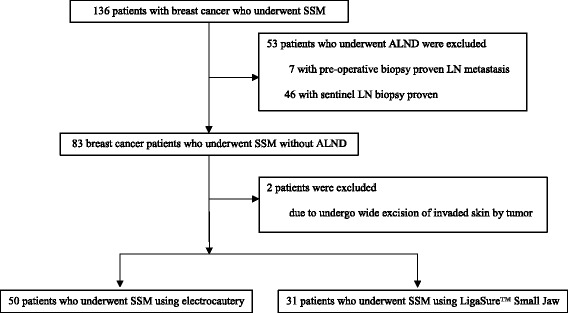
Flow chart of the study population. *SSM* skin-sparing mastectomy, *ALND* axillary lymph node dissection, *LN* lymph node

Between January 2012 and December 2015, records of 136 patients with breast cancer who underwent SSM at Korea University Medical Center, Ansan, were retrieved. Among these, 53 patients who underwent axillary lymph node dissection (ALND) for biopsy-proven metastasis were excluded to avoid differences in postoperative drainage amounts depending on whether or not ALND was performed. Two patients who underwent wide skin excision for tumor invasion by breast cancer during SSM were also excluded since these patients had a shorter operation time due to a larger operative view. Finally, 81 patients were eligible and divided into an electrocautery group and a LigaSure™ Small Jaw group, based on the devices used for SSM (Fig. 2). This retrospective study was approved by the Institutional Review Board of Korea University Medical Center, Ansan (registration number: AS16198), and the authors have no potential or actual personal, political, or financial conflicts of interest to declare.

The skin incision was made with a conventional scalpel, and subcutaneous tissue flaps were raised. The flaps were elevated to the level of the clavicle, superiorly; to the edge of the sternum, medially; to the thoraco-abdominal aponeurosis, inferiorly; and to the edge of the latissimus dorsi muscle, laterally. The dissection was continued until a complete margin of pectoral muscle and fascia bordered the superior portion of the field. Subfascial dissections were performed over the pectoralis major and over the breast, using each device.

Operative time was calculated (in minutes) from the initial skin incision to the end of bleeding control after breast removal in all cases. The total amount and days of drainage until removal and the weight of removed breast tissue were obtained from medical records. The drains were removed when the volume was less than 20 mL/day. Body mass index (BMI) was also calculated to compare the two groups. Postoperative skin necrosis requiring debridement was also analyzed.

Data were analyzed using SPSS® Statistics (version 24.0.0.0; IBM Corp., Armonk, NY, USA). Continuous variables were presented as means with standard deviations, and categorical variables were presented as numbers with percentages. The *t* test was used to compare continuous variables, and categorical variables were compared using the chi-square or Fisher’s exact test to analyze the significance of differences. Differences were considered statistically significant at a *P* value of <0.05.

## Results

### Patient and operative details

The study population included 50 patients in the electrocautery group and 31 in the LigaSure™ Small Jaw group. Mean ages were not significantly different between both groups (44.1 ± 7.5 vs. 46.4 ± 6.4 years, respectively, *P* = 0.164). BMI showed no significant difference (23.7 ± 3.3 vs. 22.7 ± 2.6, respectively, *P* = 0.173), and TNM staging did not differ between the two groups. NSM was performed in both groups, and weights of removed breast tissues were comparable between the two groups. The LigaSure™ Small Jaw group had a significantly shorter mean surgery time (117.5 ± 16.9 vs. 104.0 ± 23.6 min, respectively, *P* = 0.004) (Table 1).

**Table 1:** Comparison of patients and operative details between the two groups

	Electrocautery group (*N* = 50)	LigaSure™ Small Jaw group (*N* = 31)	*P* value
Age (years)	44.1 ± 7.5	46.4 ± 6.4	0.164
Body mass index	23.7 ± 3.3	22.7 ± 2.6	0.173
TNM stage, *N* (%)			0.171
0	15 (30.0)	10 (32.3)	
1	19 (38.0)	6 (19.4)	
2	16 (32.0)	15 (48.4)	
Nipple-sparing mastectomy, *N* (%)	22 (44.0)	13 (41.9)	0.855
Weight of removed breast (gram)	350.3 ± 155.9	412.1 ± 164.7	0.094
Operative time (min)	117.5 ± 16.9	104.0 ± 23.6	0.004

### Drainage and morbidity

Mean total volume of drainage was significantly less in the LigaSure™ Small Jaw group than in the electrocautery group (805 ± 278 vs. 694 ± 131 mL, respectively, *P* = 0.017), and the LigaSure™ Small Jaw group also had a shorter mean duration of drainage (11.3 ± 2.5 vs. 10.1 ± 2.0 days, respectively, *P* = 0.029). Regarding complications, 12 (24.0%) and 4 (12.9%) patients in both groups experienced postoperative skin necrosis requiring debridement, respectively; no differences were found between the two groups (*P* = 0.264). Postoperative seroma and hematoma formation also showed no significant difference (Table 2).

**Table 2:** Comparison of drainage and morbidity details between the two groups

	Electrocautery group (*N* = 50)	LigaSure™ Small Jaw group (*N* = 31)	*P* value
Total volume of drainage (mL)	805 ± 278	694 ± 131	0.017
Duration of drainage (days)	11.3 ± 2.5	10.1 ± 2.0	0.029
Complications			
Skin necrosis, *N* (%)	12 (24.0)	4 (12.9)	0.264
Seroma, *N* (%)	7 (14.0)	3 (9.7)	0.734
Hematoma, *N* (%)	3 (6.0)	0 (0.0)	0.282

## Discussion

SSM requires more scrupulous surgical technique than conventional total mastectomy since the entire breast has to be removed through a small incision and the skin flap has to be protected from ischemia [10]. Electrocautery is the most common device used in conventional total mastectomy, but this is known to be associated with complications such as hematomas, seromas, and prolonged drainage [11, 12]. When performing SSM, because subdermal vessels are not clearly seen, the use of electrocautery without vessel sealing could result in more frequent bleeding.

Use of the LigaSure™ Small Jaw has been studied in thyroid surgery and is known to shorten the operative time and reduce complications [13–15]. In breast surgery, several studies examined the use of LigaSure™ but employed the long type, which is used in endoscopic or open abdominal surgery [6, 7, 16]. Long-type LigaSure™ is good at sealing vessels but makes the operation inconvenient due to the longer distance from the patient. LigaSure™ Small Jaw is shorter than a previous device and could be very useful in breast surgery. This is the first report of the use of LigaSure™ Small Jaw in SSM.

Several previous studies on the use of tissue-sealing devices during conventional total mastectomy showed mean operative times comparable to those with use of electrocautery [17–19]. In contrast, our results suggested that patients in the LigaSure™ Small Jaw group had a shorter operative time than those in the electrocautery group. This might be due to reduced bleeding with sealing of subdermal vessels before dissection. In SSM, more time is needed to control a bleeding focus due to the small operative view compared with that in conventional total mastectomy; thus, reduction of bleeding could have influenced operative time in our study.

The present study showed that the use of LigaSure™ Small Jaw could significantly reduce total volume and duration of drainage in SSM. The reduction of drainage resulted from adequate sealing of lymphatics by electrothermal energy and pressure [18, 20]. We excluded patients who underwent ALND since volume and duration of drainage are easily affected, depending on whether ALND is performed. Nevertheless, we found that patients in the LigaSure™ Small Jaw group had a smaller volume and shorter duration of drainage, which might be because even subdermal lymphatics were properly sealed with this device.

The rate of flap necrosis is known to be decreased by the use of energy devices such as LigaSure™ or Harmonic® scalpel in conventional total mastectomy [18, 19, 21, 22]. Flap necrosis is associated with lateral thermal spread from the device used during the operation. Sutton et al. showed that electrocautery had the highest mean temperature and the widest thermal spread [23]. Although our result presented no significant difference in skin necrosis requiring debridement, patients in the electrocautery group tended to experience more postoperative skin necrosis requiring debridement in our study (24 vs. 12.9%, respectively).

The limitations of our study include its retrospective nature, which has the potential for bias. Unequal numbers between the two groups and the small subject numbers could be insufficient to support our results. A selection bias was unavoidable since the choice of using LigaSure™ Small Jaw was made by the patient. LigaSure™ Small Jaw has been increasingly used in breast surgery since its approval of public insurance about 2 years ago. Despite the limitations, the results of this study have clinical significance and provide important insights into the use of LigaSure™ Small Jaw in SSM.

## Conclusion

In conclusion, the use of LigaSure™ Small Jaw during SSM was associated with reduced operative time, total volume of drainage, and duration of drainage. Nevertheless, further prospective investigation is warranted to elucidate the association between operative devices and intraoperative/postoperative parameters in patients undergoing SSM.
